# The stethoscope: celebration or cremation after 200 years?

**DOI:** 10.1007/s12471-016-0826-6

**Published:** 2016-04-06

**Authors:** E. E. van der Wall

**Affiliations:** Netherlands Society of Cardiology/Holland Heart House, Moreelsepark 1, 3511 EP Utrecht, the Netherlands

The stethoscope is currently at the crossroads. For two centuries now, the stethoscope has been a central part of examining the thorax. Nevertheless, this omnipresent tool of the medical profession (‘conversation piece’) is at the heart of the debate over how diagnostic medicine should be practised.

At the moment, there are many divergent views on the value of using the stethoscope as a primary diagnostic tool for clinical investigation [1–3]. “The stethoscope is dead and its time has gone”, according to Dr. Jagat Narula, cardiologist at the Icahn School of Medicine at Mount Sinai Hospital in New York (Washington Post, 2 January 2016). This view is opposed by Dr. W. Reid Thompson, paediatrician at Johns Hopkins University School of Medicine (Baltimore, USA), who clearly stated that listening to sounds of the body will remain valuable.

Since its introduction in 1816, the stethoscope has been an indispensable bedside approach for listening to heart sounds. The French physician Dr. René Laennec, working at Necker-Enfants Malades Hospital in Paris, can be considered the ‘father’ of the stethoscope. At that time, Laennec introduced a cylindrical device, open at each end, to auscultate the thorax. He called his instrument a stethoscope, whose name is derived from the Greek words stethos, i. e. chest, and skopeo i. e. to look at (observe). Over the years, the cylindrical stethoscope was modified – after the introduction of rubber – by a device that had hearing pieces fitting into the physician’s ears. Other advancements were the introduction of the bell to recognise low-pitched sounds, and the diaphragm in order to better perceive high-pitched sounds.

Recently, the emergence of the electronic stethoscope has paved the way for a new field of computer-aided auscultation. The sounds which the stethoscope transmits from the heart, lungs, blood vessels and bowels can now be digitised, amplified, filtered and recorded. Algorithms already exist that can analyse the clues picked up by a stethoscope and offer a possible diagnosis. The newest versions of the electronic stethoscope combine ambient noise reduction technology and frictional noise dampening features with amplification, Bluetooth® technology, and an all-new user interface, for the next level of performance and ease of use, which is particularly of importance in the era of telemedicine. Several months ago, the Food and Drug Administration (FDA) approved a stethoscope that can faithfully reproduce those sounds on a cell phone application (app) thousands of miles away or send them directly to an electronic medical record. The stethoscope, called the Eko Core ($299), records the sounds of a patient’s heart and transmits them to an iPhone app. This opens the potential for heart sounds to be stored in the cloud, for clinicians to reference or analyse the data to increase their understanding of the heart.

A next major issue was the clinical introduction of ultrasound technology, which has provided the ability to visualise both anatomic structures of the heart and to assess myocardial function [4–6]. In particular, handheld ultrasound (HHU) devices, which can fit into the pocket of a physician’s white coat, have recently demonstrated the ability to make more accurate diagnoses at the bedside when compared with standard examination using the stethoscope [7–9]. Mehta et al. [10] reported in 2014 that an HHU approach provides a more accurate diagnosis than physical examination for the majority of common cardiovascular abnormalities. In that study an HHU device was used (Vscan, GE Healthcare, Milwaukee, Wisconsin) providing B‑mode and colour Doppler images but no spectral Doppler data, having a retail price of $ 7900. The development of pocket-size ultrasound devices are therefore raising questions about why physicians and medical personnel continue to wear stethoscopes around their necks [11].

Whether all this represents the rebirth of a diagnostic possibility or the soft cremation of an obsolete device has become the subject of a lively discussion in cardiology. Will the stethoscope also undergo the same fate as phonocardiography, given that HHU devices have now become available to better define cardiac anatomy, valve structures, ventricular function, and pathophysiology? Although replacement of our classical hearing device might seem at hand, there are still several reasons to withhold the cremation of the stethoscope right now.

First, physicians should become well-trained in using HHU devices. At present, HHU is not an integral part of the curriculum for the cardiologist in training. According to Metha et al. [10], attempts at introducing HHU to practising physicians have failed for several reasons such as 1) the reluctance of most physicians to obtain additional training in the use of HHU, 2) the perception that HHU examination takes considerably more time than physical examination, 3) absence of financial or other incentives; it takes more time without providing additional compensation, 4) at least among some cardiologists, there may be a concern that it will reduce the need for a standard echocardiogram, which may adversely affect their income in the current fee-for-service setting, 5) the opposite concern that, based on HHU, spurious echocardiograms will be ordered, increasing overall cost and 6) HHU is not as robust and accurate as a standard echocardiogram for confirming or excluding cardiac findings.

Second, it remains necessary to use the stethoscope for pulmonary examination and for auscultation of the abdomen to hear bowel sounds and bruits. Auscultation of the lungs is still necessary to assess the presence or absence of early stages of heart failure. The ability to auscultate with the same device will provide a truly comprehensive cardiovascular examination. These devices could have apps that would allow physicians to access Internet-based information.

Third, at present the handheld devices are very expensive when compared with the cost of a stethoscope. This might be partly counterbalanced by the greater diagnostic yield of HHU technology, avoiding sequential investigations.

Fourth, according to Silverman [12], HHU is often carried out inexpertly, is very operator-dependent, and currently cannot be hard copied into the electronic medical record documentation. One therefore needs to study in which patients the HHU replaces the stethoscope, when it augments the bedside examination, and with which symptoms and diseases it improves our diagnostic skills and influences disease management. With these data in hand, one may have guidelines for practice that will inform the cardiologist when he/she should carry out a thorough cardiac examination or he/she should reach for the HHU.

Lastly, the word stethoscope for the classical instrument is in fact a misnomer. It should have been called stethophone (from the Greek word phone = sound) at the very start. As said before, the strict meaning of stethoscope is looking at (observing) the chest. In that sense, an HHU is also a ‘stethoscope’!

Therefore, after 200 years it is time to celebrate the stethoscope (our most impressive necklace!) and to provisionally postpone its cremation until the above-mentioned issues have been resolved. The time for only listening to the heart might reach its end, but for evaluation of the heart one will always need a ‘stethoscope’, be it electronic and/or combined with ultrasound technology.
